# Animal-Assisted Therapy for Reducing Anxiety in Vulnerable Clinical Populations: A Systematic Review

**DOI:** 10.3390/healthcare14020260

**Published:** 2026-01-21

**Authors:** Nazaret Hernández-Espeso, Laura Durbán Bronchud, Gloria Bernabé-Valero

**Affiliations:** Faculty of Psychology, Catholic University of Valencia San Vicente Mártir, Av. De La Ilustración, 2, Burjassot, 46100 Valencia, Spain; laura.durban@mail.ucv.es (L.D.B.); gloria.bernabe@ucv.es (G.B.-V.)

**Keywords:** anxiety, mental health, animal-assisted therapy, clinical populations, chronic illness, disability, hospitalisation, systematic review

## Abstract

**Background**: Anxiety is highly prevalent among individuals living with disability, chronic illness, or hospitalisation, yet it often remains insufficiently addressed in healthcare settings. Animal-assisted therapy (AAT) has been proposed as a complementary intervention to reduce anxiety; however, existing evidence is fragmented across populations and methodologies. **Methods**: A systematic review was conducted following PRISMA 2020 guidelines. The review protocol was registered in PROSPERO (CRD42024494109); no amendments were made to the protocol after registration. Four databases (Scopus, APA PsycInfo, Web of Science, and PubMed) were searched for empirical studies (2013–2023) evaluating AAT delivered by trained professionals using domesticated species and reporting anxiety outcomes in individuals with disability, illness, or hospitalisation. **Results**: Thirty-one studies met eligibility criteria and were included in the review. Across heterogeneous designs, most interventions—primarily using dogs or horses—reported significant post-intervention reductions in anxiety. Randomised clinical trials consistently showed superior results compared with control conditions. AAT demonstrated beneficial effects across populations including PTSD, paediatric hospitalisation, chronic illness, disability, acute care, and trauma exposure. Long-term outcomes were mixed, and methodological variability limited comparability across studies. **Conclusions**: AAT appears to be a promising complementary intervention for anxiety management within clinical, psychosocial, and healthcare settings. Evidence supports short-term anxiolytic effects across diverse populations, although standardisation and long-term evaluations remain insufficient. Future research should establish optimal intervention parameters, mechanisms of action, and strategies for integrating AAT into multidisciplinary mental healthcare.

## 1. Introduction

Anxiety disorders are among the most prevalent mental health conditions worldwide, affecting daily functioning and overall wellbeing [1]. Their prevalence has increased in recent years, particularly during and following the COVID-19 pandemic, which was associated with a substantial rise in psychological distress worldwide [1,2]. Individuals with chronic illness, disability, neurodevelopmental conditions, or prolonged hospitalisation are especially vulnerable to heightened anxiety [3], highlighting the need for accessible, complementary interventions.

Within this context, research on human–animal interaction (HAI) has grown steadily. Evidence indicates that structured interactions with animals can support emotional regulation, reduce stress responses, and enhance engagement in therapeutic activities [4,5]. Animal-Assisted Interventions (AAI) encompass practices that integrate trained animals into therapeutic or supportive environments. Animal-Assisted Therapy (AAT), also referred to as Animal-Assisted Treatment (AATx), describes goal-directed interventions delivered by professionals with formal training and responsibility for the structured facilitation of animal-assisted interventions, as defined by international consensus guidelines. This terminology is used for methodological consistency and does not imply that intervention effects depend on specific clinical-level qualifications [6,7]. In line with recent international consensus recommendations, this review adopts the term AAT as the primary term, acknowledging that AAT and AATx are considered equivalent in the current literature [7]. Studies suggest that AAT may improve anxiety, mood, and general wellbeing across diverse populations [8,9]. Understanding the effectiveness of AAT for anxiety is highly relevant for healthcare and psychosocial services seeking non-pharmacological, person-centred strategies to support emotional wellbeing, reduce stress responses, and improve the overall care experience across diverse clinical and supportive settings. The theoretical basis for AAI is often linked to the biophilia hypothesis, which proposes an innate human inclination to connect with other living beings [10]. Positive interactions with animals have been associated with improved emotional states and reductions in physiological stress markers [11,12], supporting the potential of AAT for anxiety reduction.

Despite promising findings, research in this field shows substantial methodological variability, particularly regarding species incorporated in AAT, intervention duration, session structure, and outcome measures [13]. Additionally, some studies have included wild or non-domesticated species, which conflicts with international welfare guidelines. According to IAHAIO standards [6], AAI should involve only domesticated or appropriately domesticated species to ensure welfare, safety, and methodological consistency.

These limitations underscore the need for a systematic synthesis of AAT interventions targeting anxiety and conducted with domesticated species under recognised professional standards. This review consolidates current evidence on such interventions in individuals experiencing disability, illness, or hospitalisation, aiming to clarify existing knowledge and support the development of ethical and effective practice within the HAI field.

In this context, anxiety represents a central psychological condition that can significantly affect emotional wellbeing and recovery in medically vulnerable populations. Although animal-assisted interventions have been applied across a range of clinical and healthcare settings, evidence regarding their effectiveness in reducing anxiety remains dispersed. The present review therefore examines the effectiveness of AAT in reducing anxiety among individuals living with disability, illness, or hospitalisation, and summarises how such interventions have been implemented in practice.

### Objectives

The aim of this study is to provide empirical evidence regarding the effectiveness of animal-assisted therapy (AAT) in mitigating anxiety associated with disability, illness, or hospitalisation. The specific objectives are: (1) analyse the methodological characteristics of AAT interventions in relation to anxiety, including the type of animal incorporated, the activities performed with the animal, the timing and number of sessions, and the assessment instruments used; (2) in addition, analyse the research designs of the studies; (3) assess the effectiveness of AAT in reducing anxiety in different populations of people with disabilities, illnesses, or hospital admissions.

## 2. Materials and Methods

Based on the PRISMA 2020 statement [14], this section describes the methodological process followed in this systematic review. The purpose of the methodological approach was not only to identify existing interventions but also to examine how AAT has been implemented in clinical, educational, psychosocial, and healthcare contexts to reduce anxiety. This systematic review was prospectively registered in the International Prospective Register of Systematic Reviews (PROSPERO) under the registration number CRD42024494109. All methods were specified a priori and followed PRISMA 2020 guidelines [14].

### 2.1. Search Procedure and Eligibility Criteria

The search strategy was developed following the PICO framework, which guided the formulation of the search equation and the definition of eligibility criteria. Table 1 presents the application of the PICO strategy to this review and the criteria used for selecting studies.

To ensure methodological and ethical rigour, clear inclusion and exclusion criteria were applied during the study selection process. Only empirical studies evaluating structured Animal-Assisted Therapy (AAT) interventions delivered by trained professionals and reporting anxiety-related outcomes were included. Eligible study designs comprised randomised controlled trials, quasi-experimental studies, pilot studies, and other empirical quantitative designs. Studies were excluded if they did not constitute AAT interventions, did not report anxiety outcomes, were theoretical or review papers, qualitative studies, case series, dissertations, or involved wild or non-domesticated species. The exclusion of non-domesticated animals was conducted in accordance with IAHAIO guidelines [6], which emphasise animal welfare, participant safety, and methodological consistency.

### 2.2. Evaluating the Quality of Studies

The levels of scientific evidence proposed by Sánchez-Meca and Botella [15] were used to guide the inclusion of studies with sufficient methodological quality. Eligible designs included randomised controlled trials, quasi-experimental studies (with or without control groups), pre–post designs, and pilot studies. Lower-quality evidence (e.g., case series or expert opinion) was excluded. A formal risk of bias assessment using standardised tools was not conducted, which is acknowledged as a limitation of this review. Importantly, these criteria were applied exclusively to determine study eligibility during the selection process and were not used to weight findings or rank studies according to methodological quality.

### 2.3. Information Sources

Articles were retrieved from four major databases: Scopus, APA PsycInfo, Web of Science, and PubMed. The search was conducted between 4 December and 20 December 2023.

### 2.4. Search Strategy

An initial exploratory search was conducted to refine keywords and assess the specificity required for the search equation. Consultation of the APA PsycInfo Thesaurus and Word Reference helped identify appropriate term variants. Preliminary terms included ‘therapy,’ ‘animal,’ and ‘anxiety,’ supplemented with synonyms and specific therapy-related descriptors (e.g., equine therapy, dog therapy) to capture studies that might not be indexed under broader AAT terminology.

All terms were combined using Boolean operators to construct the final search equation:

(animal therapy OR pet therapy OR dog therapy OR animal assisted therapy OR AAT OR therapy dog OR hippotherapy OR equine therapy OR horse therapy OR pet therapy OR interspecies interaction OR animal facilitated therapy OR pet facilitated therapy OR pet-assisted therapy) AND (anxiety OR anxiousness OR nervousness OR worry OR anxiously OR anxiety disorder OR anxiety disorder generalised OR panic attack OR panic disorder OR phobias OR selective mutism OR separation anxiety disorder).

A temporal filter restricted results to studies published within the last 10 years to ensure contemporary relevance while allowing sufficient coverage of existing evidence.

### 2.5. Data Selection and Collection Process

The Rayyan tool (Rayyan Systems Inc., Cambridge, MA, USA) [16] was used to manage and screen all records. Three researchers independently screened titles and abstracts in blinded mode, identifying included, excluded, questionable, or conflicting records. Conflicts were resolved through discussion, based on study objectives and eligibility criteria.

Consensus was reached on six categories of topics to exclude: “pets” (dog–owner interaction not involving AAT), “assistance dogs,” “other disorders” (AAT not involving anxiety), “informational noise” (articles in neuroscience, psychopharmacology, mindfulness, medicine, etc., not related to AAT), “veterinary,” and “human–animal interaction without AAT methodology.” Population labels were standardised across studies as follows: “patients,” “children,” “adults,” “dental,” “workers,” and “students.” Animals were categorised using labels such as ‘dogs’, ‘horses’, and other initially screened categories; however, consistent with IAHAIO guidelines [6], only studies involving domesticated species were included in the final synthesis. Following the PICO criteria, full texts were screened, and studies meeting all criteria were selected. A total of 31 studies were retained for qualitative synthesis.

## 3. Synthesis Methods

The synthesis of results was performed after thorough reading of the 31 selected studies. Study characteristics were categorised manually and presented in Table 2. Findings were summarised thematically across three domains: (1) methodological characteristics of AAT interventions (species, frequency, session duration, activities); (2) descriptive and research characteristics (population types, study design, publication dates, sample size, and country); and (3) effectiveness of AAT in reducing anxiety.

## 4. Results

The main features of the 31 selected studies are presented below. Results are organised into methodological characteristics of the interventions, population descriptors, and findings related to anxiety reduction.

### 4.1. Selection of Studies

**Figure 1:**
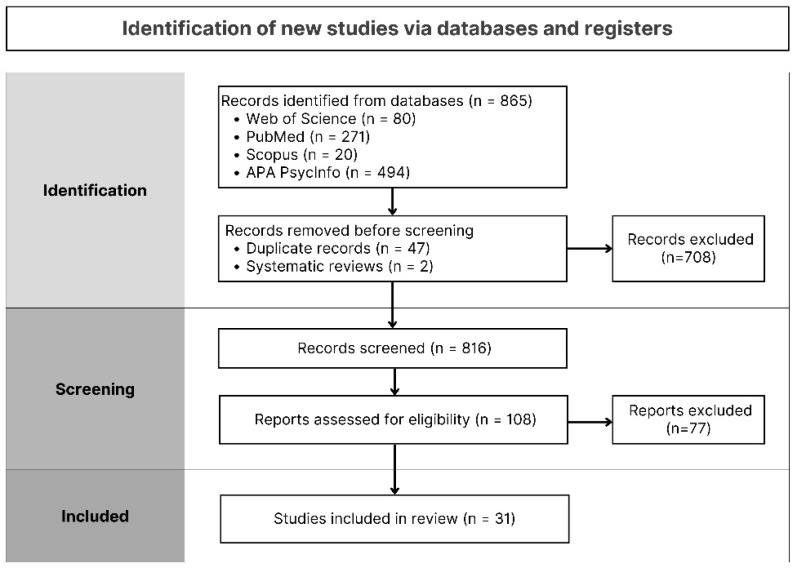
Flow diagram. Note. Flow diagram illustrating the study selection process, adapted from PRISMA 2020 [14].

A total of 865 results were generated from the databases. After removing duplicates and systematic reviews, 816 records remained. These were initially screened for relevance based on their titles and abstracts. Entries unrelated to AAT, lacking complete publication, or failing to address anxiety symptoms were excluded (n = 708). The full texts of the remaining 108 articles were then thoroughly assessed, resulting in the exclusion of 77 articles that did not adequately address the research question. Following title, abstract, and full-text screening, a total of thirty-one studies met the eligibility criteria and were included in the review (see Figure 1).

### 4.2. Methodological Characteristics of Animal-Assisted Therapy Interventions

The main features of the 31 selected articles are described below (see Table 2).

The species most frequently incorporated in AAT interventions were dogs (n = 18), followed by horses (n = 12), and, to a much lesser extent, cats (n = 1). No studies involving wild or non-domesticated animals were included, in accordance with IAHAIO guidelines [6].

Intervention duration varied considerably across studies. Among the 18 studies reporting this information, 33.3% lasted 1 week (n = 6), 16.7% lasted 4–6 weeks (n = 3), another 16.7% ranged up to 8 weeks (n = 3), 22.2% spanned 12–16 weeks (n = 4), and 11.1% extended from 32 to 48 weeks (n = 2). Among the 27 studies providing session-frequency data, 22.2% delivered between 1 and 2 sessions (n = 6), 25.9% delivered 3–6 sessions (n = 7), 22.2% delivered 7–8 sessions (n = 6), 11.1% delivered 12–14 sessions (n = 3), 7.4% delivered 16–20 sessions (n = 2), 7.4% delivered 32–48 sessions (n = 2), and 3.7% delivered a total of 74 sessions (n = 1).

Session duration also varied. Among the 28 studies reporting this parameter, 28.6% delivered sessions of 5–15 min (n = 8), 35.7% delivered 20–40 min (n = 10), 25.0% delivered 45–60 min (n = 7), 10.7% delivered 90-min sessions (n = 3), and 3.6% delivered sessions lasting 120, 180, or 240 min (n = 1 each). In analysing the activities conducted during the sessions, particularly in the case of the 18 studies involving dogs, among the respondents (n = 15), 73.33% (n = 11) proposed petting the dog, 33.33% (n = 5) talked about the dog (or the patient’s pets) or performed care activities (brushing, feeding, etc.). Training activities were described in 26.66% (n = 4) of studies, and 13.33% (n = 2) included playing with the dog or walking it on a leash. Only one study (6.66%, n = 1) incorporated giving treats to the dog. Furthermore, one study uniquely reported a broader range of activities, including talking to the dog, conducting agility exercises, using the dog for emotional support during medical appointments, learning about canine needs and behaviour (ethology), and simply spending time in the dog’s presence without engaging in specific activities.

In the 12 studies involving horses, 41.66% (n = 5) reported horse care activities, while 50% (n = 6) focused on equitation and riding tasks. Ground handling and guiding horses through obstacle courses were proposed in 16.66% (n = 2) of studies. Notably, only one study (8.3%, n = 1) mentioned engaging in activities such as walking with the horses, non-verbal communication, or putting the bridle on the horse’s head. In the only study involving a cat, the activities performed with the animal were not specified.

### 4.3. Descriptive and Research Characteristics of Study Designs

The socio-demographic and typological characteristics of the 31 studies included in this review are summarised below. Most studies (n = 20) examined adult populations, while 10 focused on children and adolescents, and one study included both adults and children [41]. The most extensively studied population across the sample consisted of individuals diagnosed with post-traumatic stress disorder (PTSD) (n = 10). This was followed by studies involving diverse populations with smaller research representation, including paediatric samples (n = 7), adults with trauma of varying severity (n = 4), individuals experiencing pain (n = 3), and participants with disabilities (n = 2). Other groups—such as individuals with severe mental illness, dementia, systemic sclerosis, cancer, or addictive behaviours—were each represented in only one study.

With respect to research design, seven studies employed randomised clinical trials including both a control group and random assignment. Seven quasi-experimental studies were identified, four of which used pre-post measurements and control groups but lacked randomisation (prospective non-randomised controlled studies). One quasi-experimental study included a control group without pre–post assessment, and one used a comparison design with a non-equivalent group of healthy participants. Additionally, one study included randomisation but did not compare the intervention and control groups. The review also identified 11 pre-experimental studies with pre-post measurements but no control group, five pilot studies employing within-subject designs, and two descriptive studies that relied exclusively on qualitative methodology.

Publication dates ranged from 2014 to 2023, with a concentration of studies published in 2018 (n = 6), 2021 (n = 5), and 2023 (n = 6). Most research was conducted in the United States (n = 19). Other countries with fewer publications included Canada (n = 2), Spain (n = 2), and Germany, Sweden, Switzerland, Brazil, Turkey, Italy, Finland, and South Korea, each represented by a single study.

Sample sizes varied considerably across studies. The two largest studies included 334 participants (Spain) and 218 participants (USA). Five studies involved samples of approximately 100 participants, twelve included around 50 participants, nine had approximately 20 participants, and four studies included fewer than 10 participants.

### 4.4. Effectiveness of Animal-Assisted Therapy in Reducing Concurrent Anxiety

From a clinical perspective, the evidence consistently indicates that AAT is associated with reductions in anxiety across diverse populations, despite heterogeneity in study designs and intervention characteristics. This section describes the instruments used in the primary studies to assess anxiety. The 31 studies included in this review employed a wide range of assessment tools, some of which measured anxiety exclusively (e.g., the State–Trait Anxiety Inventory, STAI), while others captured anxiety as part of broader symptomatology. For example, the PTSD Checklist for DSM-5 (PCL-5) assesses the severity of post-traumatic stress disorder [32], including symptoms such as anxiety, flashbacks, and emotional numbing [17].

The STAI and its adaptations were the most frequently used instruments, appearing in 32% of studies (n = 10). The Post-Traumatic Stress Disorder Checklist (PCL) and its adaptations were used in 23% of studies (n = 7). Developed by Spielberger et al. [48], the STAI is considered an optimal tool for detecting emotional changes targeted by AAT interventions. Similarly, the PCL [49] has demonstrated strong diagnostic efficiency for evaluating anxiety- and stress-related symptoms [50]. The use of standardised and validated instruments across studies enhances the comparability and interpretability of anxiety outcomes. Other instruments used included the Generalized Anxiety Disorder scale (GAD-7) in 9.37% of studies (n = 3), and both the Burns Anxiety Inventory (BAI) and the Perceived Stress Scale (PSS) in 6.25% of studies (n = 2 each). Several additional measures appeared in only one of the 31 studies, including the Numeric Rating Scale of Anxiety (NRS), the Hopkins Symptom Checklist-25 (HSCL-25), the Edmonton Symptom Assessment System–Revised (ESAS-R), the Social Anxiety in Children Scale–Revised (SACS-R), the Social Phobia Scale (SPS), the Patient Health Questionnaire (PHQ-9), and the Korean Experiences in Close Relationships Scale–Revised (K-ECRS).

Broader emotional and stress-related measures were also used, such as the Depression Anxiety Stress Scale–21 (DASS-21), the Child Stress Symptoms Inventory, the Cornell Scale for Depression in Dementia (CSDD) anxiety subtest, the Modified Yale Preoperative Anxiety Scale (m-YPAS), the Beck Depression Inventory (R-BDI), and semi-structured interviews conducted via Zoom (Zoom Video Communications, Inc., San Jose, CA, USA). Physiological indicators were collected in 21.87% of studies (n = 7), including heart rate and respiratory rate. Cortisol levels measured through oral swabs were collected in 6.25% of studies (n = 2), and another 6.25% (n = 2) used self-developed questionnaires to assess anxiety. Depression was also examined as a secondary variable associated with anxiety in 14.8% of studies (n = 4), measured using the Beck Depression Inventory (BDI) or its derivatives. Although the R-BDI includes an item on anxiety, this item is not used in the calculation of the depression index.

Regarding the impact of AAT on reducing anxiety associated with disability, illness, or hospitalisation, the majority of studies reported significant reductions in anxiety symptoms following the intervention. These decreases were observed in both intragroup (pre–post) and intergroup (experimental vs. control group) comparisons. Furthermore, several studies indicated that these improvements were maintained over time. Three studies [23,25,39] found that reductions in anxiety persisted in both short- and long-term follow-up assessments. In contrast, two studies with follow-up evaluations [17,44] reported that improvements in anxiety were temporary and not sustained.

Across all seven randomised controlled trials (RCTs), which are often considered a gold standard for evaluating intervention effectiveness but may not always be fully suited to complex experiential interventions, participants in the experimental groups consistently experienced significant reductions in anxiety and stress compared with control groups. This reinforces the positive impact of AAT within rigorous research designs. Among the seven quasi-experimental studies, three demonstrated clear decreases in anxiety in the intervention group compared with controls, while the remaining four showed more modest or mixed findings. For example, Hinic et al. [28] found that anxiety levels decreased in both the AAT and control (puzzle-building) conditions, although the reduction was greater among children interacting with the dog. Similarly, Silva and Osório [46] observed decreased anxiety among caregivers of children with leukaemia, though the children’s own anxiety levels did not significantly change—a result that may be attributable to the use of a single anxiety indicator. In the study by Kang et al. [34], both the experimental group (children with gambling addiction) and the control group (healthy children) showed reductions in anxious behaviour.

Among the 11 pre-experimental studies without control groups, 10 reported significant pre–post reductions in anxiety, whereas one study [20] did not find significant changes, despite observing medium effect-size improvements in both anxiety and depression. Qualitative studies also supported the anxiolytic effects of AAT. Johnson et al. [33] described participants feeling relaxed during AAT sessions, while Shelton [45] identified “Anxiety” as a key theme, with subthemes such as Insecure Attachment, Fear of Falling Short of Expectations, and Abandonment.

When examining effectiveness across specific populations, evidence suggests that AAT benefits all groups studied. AAT effectively reduced anxiety among individuals with PTSD [25,33,37], although several studies [17,19,24,32,40,42,44] highlighted uncertainty regarding the long-term stability of these effects. In populations with severe mental illness, studies [20,29] reported significant reductions in anxiety. Similar benefits were observed among hospitalised patients—both adults [22,28] and children or adolescents [18,35,36,39,41,43,46]. Reductions in anxiety were also documented in individuals with disabilities or chronic medical conditions, both in adults [21,26,27,47] and in children [23]. Likewise, AAT demonstrated positive effects among individuals with diverse trauma experiences, including adults [31] and adolescents [34]. Collectively, these findings suggest that AAT can be an effective option for reducing anxiety across a broad range of populations and age groups, demonstrating patterns of benefit that are consistent with those reported in other experiential and psychosocial interventions.

Some studies also suggest potential gender differences in treatment response. Carey et al. [21] and Giuliani & Jacquemettaz [27] reported greater reductions in anxiety among male participants compared with females, indicating a possible moderating effect of gender.

Two studies directly compared AAT with Cognitive Behavioural Therapy (CBT) in female prisoners with severe mental disorders. In Holman et al. [30], both treatment groups—CBT and AAT—showed significant reductions in anxiety. Although CBT was considered more cost-effective and logistically simpler, the authors proposed that combining CBT with therapies such as AAT may yield more durable long-term effects.

Finally, with regard to the influence of treatment characteristics on outcomes, the reviewed studies did not identify specific parameters (e.g., intervention length in weeks, number or duration of sessions, or type of animal) that consistently produced superior results. Each intervention was tailored to the study’s objectives, population needs, and characteristics of the animals involved. Therefore, these variables do not appear to fully account for the variability observed in anxiety reduction outcomes.

## 5. Discussion

The findings of this review indicate that animal-assisted therapy (AAT) shows consistent promise as a complementary intervention for reducing anxiety across diverse clinical and healthcare contexts. Importantly, these results highlight the potential value of AAT as an adjunctive tool in populations where conventional psychological approaches may be limited by physical, medical, developmental, or contextual constraints. Despite substantial variability in designs, treatment structures, and species incorporated, the overall pattern of evidence suggests that AAT reliably produces short-term reductions in anxiety, with some studies also reporting longer-term benefits. These patterns of benefit are consistent with findings reported across a range of experiential and relational interventions, suggesting that AAT reflects context-specific applications of mechanisms observed more broadly in experiential therapy research. These outcomes align with broader psychological research demonstrating that supportive, emotionally salient interactions can modulate stress and anxiety responses [10,11].

Although AAT has been widely applied across clinical, educational, and community contexts, interventions designed specifically to reduce anxiety in medically or psychologically vulnerable populations remain methodologically diverse. This heterogeneity reflects both the rapid expansion of the field and the absence of unified clinical guidelines for AAT implementation. The present review therefore contributes to the psychological and health literature by consolidating how AAT programmes have been operationalised—highlighting common patterns in species selection, session structure, and therapeutic activities—and by clarifying their effectiveness across populations experiencing disability, illness, or hospitalisation. Taken together, these observations provide a foundation for interpreting the more detailed patterns emerging across diagnostic categories and study designs.

From a clinical perspective, these findings are particularly relevant for healthcare and psychological practitioners seeking complementary interventions that are feasible within constrained environments, such as inpatient units, rehabilitation services, or trauma-focused settings. Individuals in these contexts often face barriers that limit access to conventional psychological treatments, including physical discomfort, medical procedures, cognitive overload, or decreased emotional engagement. AAT may help bridge this gap by providing a non-threatening, motivating stimulus that facilitates emotional regulation, enhances engagement, and reduces physiological arousal, thereby increasing receptivity to parallel treatments [4,5]. Importantly, the evidence reviewed does not allow attribution of anxiolytic effects to specific professional credentials or clinical skills. Across studies, outcomes appear more closely associated with the structured human–animal interaction itself and the practitioner’s role in facilitating engagement, rather than with the application of formal psychotherapeutic techniques. Notably, several of the activities described across the included studies involve experiential and relational components that do not inherently require clinical-level skills, further supporting the interpretation of the practitioner’s role as one of structured facilitation rather than clinical treatment delivery.

A key finding of this review is the consistency of positive outcomes across heterogeneous populations, including individuals with PTSD [25,33,37], hospitalised adults [22,28], hospitalised children and adolescents [18,35,36,39,41,43,46], individuals with chronic illnesses or disabilities [21,26,27,51], and populations experiencing trauma [31,34]. Despite substantial methodological variability, the collective evidence suggests that AAT can serve as a useful complementary intervention for managing anxiety symptoms across settings and life stages. Such consistency across diverse populations mirrors patterns commonly reported in experiential and psychosocial intervention research, suggesting that these findings reflect mechanisms shared across relational and experiential approaches rather than effects unique to AAT.

Long-term effects, however, remain mixed. Some studies found that reductions in anxiety were sustained at follow-up [23,25,39], while others reported only temporary improvements [17,44]. This inconsistency suggests that the durability of AAT effects may depend on population characteristics, intervention intensity, or the presence of concurrent therapies, highlighting the need for long-term longitudinal research. The reasons for these discrepant follow-up findings remain unclear. Future research should examine whether differences in population characteristics, intervention intensity, duration, follow-up length, or concurrent treatments may account for the variability in the persistence of anxiety reductions. Debates within the wilderness and experiential therapy literature have highlighted that long-term outcomes may be sensitive to factors such as motivation for change, referral context, and selective outcome reporting, suggesting that sustained effects are best understood through longitudinal, theory-informed, and context-aware approaches rather than single outcome indicators [52].

Differences also emerged when considering study design. All seven randomised controlled trials reported significant reductions in anxiety in the experimental groups compared with controls, reinforcing the robustness of these findings. In contrast, quasi-experimental designs yielded more heterogeneous results: three studies identified clear improvements (e.g., [22,44]), while others reported more nuanced outcomes [28,34,46]. For example, in Hinic et al. [28], both the puzzle-control group and the AAT group showed reductions in anxiety-state, though the decrease was greater among children interacting with the dog. Similarly, Silva and Osório [46] observed anxiety reductions in caregivers but not in paediatric patients with leukaemia, possibly due to the use of a single anxiety measure.

Among the 11 pre-experimental studies, 10 reported significant pre–post reductions, with the exception of Cappelen et al. [20], who found no statistically significant change, although effect-size estimations indicated a downward trend in both anxiety and depression. Qualitative studies also reinforced the subjective benefits of AAT, with participants reporting relaxation and emotional relief [33] and identifying themes such as insecurity, fear of failure, and abandonment as areas positively influenced by AAT [45].

Across diagnostic categories, AAT consistently reduced anxiety. However, several studies raised important questions about the persistence of improvements over time among individuals with PTSD [17,19,24,32,40,42]. This suggests that while AAT may offer immediate symptom relief, longer-term effects may require ongoing engagement or integration with other therapeutic modalities. Future research should explicitly examine why PTSD populations are frequently targeted in AAT interventions, including whether features such as heightened physiological arousal, difficulties with emotional regulation, or challenges with verbal engagement may make experiential and relational approaches particularly suitable for this group. In parallel, for clinicians, educators, rehabilitation specialists, and healthcare professionals, understanding how AAT has been applied across contexts provides a framework for selecting appropriate intervention modalities and tailoring them to the needs of specific patient groups. An ecological perspective may further help contextualise these findings. Psychological distress, including trauma-related symptoms, does not arise solely from individual factors but is embedded within broader relational, institutional, and environmental contexts. From this perspective, sustained change may be constrained when the individual’s ecology remains unchanged—a consideration that is particularly salient for children and adolescents, who often have limited autonomy over their environments. Recent ecological dynamics and human–nature relationship research emphasises the importance of considering how interventions interact with wider systems in shaping mental health outcomes [53,54,55].

In addition to these population-level effects, individual characteristics may also influence treatment responsiveness. Some evidence also points to possible gender differences [21,27], reporting greater reductions in anxiety among men than women. Related gender-associated patterns have also been discussed in wilderness and experiential therapy research, suggesting that gender may act as a potential moderating factor in experiential intervention outcomes [56]. Although preliminary, these findings highlight the need to explore potential moderating factors such as gender, attachment style, and baseline symptom severity.

Two studies comparing AAT with Cognitive Behavioural Therapy (CBT) in female prisoners with severe mental disorders [30] found significant reductions in anxiety in both intervention groups. Although CBT was considered more cost-effective and easier to implement, combining CBT with AAT was suggested to potentially produce more durable gains, emphasising the value of multimodal treatment approaches. These findings suggest that AAT may be comparable to established psychological interventions such as CBT in reducing anxiety, highlighting its potential legitimacy as a therapeutic option. Future research would benefit from extending such comparisons to other non-experiential and talk-based interventions, and from systematically examining engagement and dropout rates, as these factors may represent an important distinguishing feature of experiential approaches.

Importantly, this review found no consistent association between treatment characteristics—such as number of sessions, session length, total intervention duration, or type of animal—and the magnitude of anxiety reduction. Each intervention was tailored to the needs of its population and the specificities of the animals involved, suggesting that therapeutic outcomes may depend more on the quality of human–animal interaction than on procedural standardisation. These findings are also consistent with broader literature on experiential and outdoor therapies, which has similarly reported positive outcomes across diverse populations and intervention formats, suggesting that AAT may share common mechanisms with other relational and experiential approaches rather than representing a uniquely distinct modality [57].

Rather than viewing this methodological diversity as a limitation to be resolved, such heterogeneity may also reflect the adaptive and context-sensitive nature of AAT. From this perspective, AAT can be understood as one effective option within a broader field of established psychosocial and experiential interventions, rather than as an intervention that must be isolated or reduced to a single research paradigm. Recognising this diversity may help avoid overly reductive approaches and support more ecologically valid directions for future research.

Despite encouraging results, several limitations should be considered. Methodological heterogeneity, wide variability in sample sizes, and inconsistent use of control groups limit the generalisability of findings. The diversity of anxiety measures used across studies also complicates direct comparison. Future research would benefit from greater methodological consistency, standardised outcome measures, larger sample sizes, and longitudinal designs to assess long-term effects.

Overall, the evidence reviewed suggests that AAT is a promising intervention for reducing anxiety across diverse populations. However, future research should aim to clarify the mechanisms underlying these effects, identify moderators such as gender or diagnosis, and determine the optimal ways to integrate AAT within broader evidence-based treatment frameworks.

### 5.1. Implications for Practice

The findings of this systematic review offer several practical considerations for the design of future AAT programmes aimed at reducing anxiety. First, both dogs and horses appear to be particularly suitable for interventions across a wide range of populations affected by disability or illness. These species were the most frequently incorporated in the reviewed studies and consistently demonstrated positive effects on anxiety reduction, making them strong candidates for future therapeutic applications. Importantly, the predominance of dogs and horses in the reviewed studies should not be interpreted as evidence of their superior therapeutic effectiveness, but rather as a reflection of current research practices, cultural familiarity, and logistical feasibility. Other animal species may offer comparable benefits, and their potential effectiveness warrants systematic investigation in future research.

Second, the evidence suggests that session frequency, session duration, and overall intervention length can vary flexibly without a clearly defined threshold required for effectiveness. This flexibility allows practitioners to tailor AAT programmes according to the specific needs, contexts, and capacities of their target populations. At present, the available literature does not allow the identification of a minimum number of AAT sessions required to achieve meaningful anxiety reduction, nor does it provide consistent data on dropout following single-session interventions. This represents an important gap in the evidence base, highlighting the need for future studies to systematically examine engagement, retention, and dose–response relationships in AAT interventions.

Third, incorporating activities that involve physical contact and purposeful interaction appears to be beneficial. For dog-assisted interventions, practices such as petting, grooming, or close physical proximity were frequently associated with reductions in anxiety. Similarly, in horse-assisted interventions, activities such as riding, grooming, and stable-care tasks were among the most effective components identified in the reviewed studies. From an ecological dynamics perspective, such benefits may arise from the active engagement of individuals within meaningful relational and environmental contexts, where perception–action coupling, embodied interaction, and affective attunement may act as key mechanisms of change [53,55].

Finally, although not derived directly from the empirical findings of this review, considerations regarding animal welfare and ecological context remain essential when designing AAT programmes. From an ecological dynamics and human–environment interaction perspective, the suitability of animal species is closely linked to the quality of relational, environmental, and ethical conditions under which interactions occur. In line with international welfare guidance [6] and ecological perspectives emphasising reciprocal human–environment relationships, the use of wild or non-domesticated species in AAT should be approached with caution, ensuring that animals’ behavioural, physical, and social needs are fully respected.

For clinicians and professionals working in healthcare, psychosocial, educational, or rehabilitation settings, the findings of this review suggest that AAT may be considered a broadly applicable complementary intervention for anxiety across diverse populations and contexts. However, the current evidence does not support population-specific or parameter-specific prescriptions (e.g., species selection, session duration, or intervention length). Rather than informing tailored intervention protocols, the findings indicate that AAT interventions of varying formats and intensities may yield comparable anxiety-related benefits, underscoring the need for future research to determine whether and how population-specific adaptations may be meaningfully identified.

### 5.2. Limitations and Further Research

This systematic review has several limitations that should be considered. Most primary studies included small or medium sample sizes, which limits the generalisability of the findings. Although logistical difficulties may partly explain this, small samples (particularly in qualitative research) can still yield valuable insights. The two qualitative studies included in this review provide meaningful contributions and highlight the need for further qualitative work to better understand the mechanisms underlying AAT. Another limitation concerns methodological heterogeneity. In line with research on experiential and adventure-based interventions, the complexity, contextual sensitivity, and relational nature of AAT may limit the feasibility and interpretability of randomised controlled trials as the sole benchmark of evidence. Previous work has highlighted that RCT designs may inadequately capture key mechanisms of change in experiential interventions, supporting the value of alternative and mixed-method research approaches for understanding effectiveness, engagement, and real-world applicability [58,59]. While nearly half of the studies used quasi-experimental or randomised controlled designs (n = 14), many lacked a control group. Nonetheless, observational, pre-experimental and qualitative studies produced results largely consistent with higher-quality designs. Compared with previous reviews, the field has grown substantially, and the present review includes a greater number of studies specifically targeting anxiety. Variability in populations and intervention characteristics also poses challenges. Several groups—such as individuals with severe mental illness, dementia, systemic sclerosis, oncology patients, or addictive behaviours—were represented by only one study, preventing population-specific conclusions. Intervention duration and session structures also varied markedly across studies, limiting direct comparison. Further research should move beyond reductionist assumptions and instead seek to develop flexible, principle-based frameworks that acknowledge the diversity of populations, contexts, and intervention formats within AAT, rather than attempting to derive narrowly standardised, population-specific guidelines.

Furthermore, certain methodological limitations inherent to this review should be acknowledged. Specifically, we did not formally assess risk of reporting bias (e.g., publication bias), which may influence the completeness and representativeness of the evidence base. Likewise, no formal certainty-of-evidence assessment (e.g., GRADE) was conducted, meaning that the strength of the conclusions should be interpreted with appropriate caution. Finally, given the heterogeneity of study designs and comparators, this review cannot determine the unique contribution of AAT relative to other evidence-based treatments for anxiety. Future research should assess long-term effects, potential adverse outcomes, optimal intervention dosage, and cost–benefit considerations. From this perspective, methodological heterogeneity should be understood as a reflection of the adaptive and context-sensitive nature of AAT rather than as a deficiency of the existing evidence base.

## 6. Conclusions

This systematic review demonstrates that Animal-Assisted Therapy (AAT) is generally effective in reducing anxiety across a wide range of populations, including individuals with PTSD, hospitalised patients, children and adolescents, and people with disabilities or chronic illness. Despite substantial variability in study designs, sample sizes, and intervention structures, most studies reported significant reductions in anxiety following AAT, and in some cases these improvements were sustained over time. Although this review focused specifically on PTSD as defined within diagnostic frameworks, future research may usefully examine whether similar benefits of AAT extend to broader trauma-related experiences, including complex and socio-cultural forms of distress, and explore whether comparable experiential mechanisms are involved. Despite methodological heterogeneity and small sample sizes, which limit the generalisability of the findings, the overall pattern of evidence supports the use of AAT as an effective intervention for managing anxiety. No specific intervention parameters, such as species, duration, or session frequency, were consistently associated with better outcomes, suggesting that flexible, population-sensitive designs may be appropriate. Overall, evidence suggests that AAT is a meaningful and practically relevant intervention for anxiety across medically and psychologically vulnerable populations. To support its broader integration into psychological and healthcare practice, future research should clarify optimal intervention parameters, evaluate long-term effects, and align outcome measures with established standards in clinical anxiety assessment.

## Figures and Tables

**Table 1:** Selection criteria for studies in the eligibility phase classified according to PIOS indicators.

PIO Indicator	Study Selection Criteria
Patient Population	Inclusion - People with disabilities, illness or hospitalisation who present symptoms of anxiety.
Intervention	Inclusion - Studies that address the issue of anxiety through AAT Exclusion - Studies that look at human–animal interaction but are not animal-assisted therapy programmes (e.g., informal animal-assisted activities or interactions involving assistance dogs) - Studies involving wild animals were excluded, in accordance with IAHAIO guidelines [6]
Comparison	Inclusion - All studies comparing AAT with other treatments or with control group
Outcomes	Inclusion - Papers showing an effect on anxiety of people with disability, illness or hospitalisation through AAT - Papers published in the last ten years. - Papers in any language Exclusion - No clear results or conclusions drawn from an experimental point of view on the impact on anxiety
Study design	Inclusion - Empirical studies (Randomised Clinical Trials (RCTs), cohort studies, outcome research, ecological studies and case–control studies). Exclusion - Literature reviews - Doctoral theses and dissertations

**Table 2:** Summary of results.

Author (Year)	Country	Animal	Diagnosis	Study Design	Sample			Evaluation Tools	Duration		
					Total	Control Group	Experimental Group		Minutes	Sessions	Weeks
1 Arnon et al. (2020) [17]	USA	Horse	PTSD	Pilot study	8	-	-	PCL-5	90	8	-
2 Barker et al. (2015) [18]	USA	Dog	Paediatrics	Quasi-experimental	40	-	-	NRS	10	1	-
3 Bergen-Cico et al. (2018) [19]	USA	Dog	PTSD	Quasi-experimental (longitudinal)	48	14	34	PCL-M, PSS	90	48	48
4 Cappelen et al. (2023) [20]	Sweden	Horse	Schizophrenia	Pilot study	6	-	-	HSCL-25	240	6	12
5 Carey et al. (2022) [21]	Canada	Dog	Pain	RCT	97	-	-	ESAS-r and monitor to measure frequencies	10	-	-
6 Coakley et al. (2021) [22]	USA	Dog	Oncology and general surgery	Quasi-experimental (pre–post)	59	-	-	STAI, oral swabs, heart and respiratory rates	15	2	1
7 Demiralay & Keser (2022) [23]	Turkey	Cat	Physical disability	RCT (single-blind)	44	23	21	PSS, SACS-R and blood pressure measurements	45–60	7	**7**
8 Earles et al. (2015) [24]	USA	Horse	PTSD	Non- experimental	16	-	-	PCL-S y GAD-7	120	6	-
9 Fisher et al. (2021) [25]	USA	Horse	PTSD	Open trial	63	-	-	PCL-5	90	8	-
10 Fiori et al. (2018) [26]	USA	Dog	Systemic sclerosis	Experimental	53	-	-	STAI-S, STAI-T, SIAS y SPS	50	20	1
11 Giuliani & Jacquemettaz (2017) [27]	Sweden	Dog	Intellectual disability	Observational study	53	-	-	STAI	30	2	-
12 Hinic et al. (2019) [28]	USA	Dog	Paediatrics	Quasi-experimental	93	43	50	STAIC, parent background questionnaire	8–10	2	1
13 Holman, Ellmo, Wilkerson & Johnson (2020a) [29]	USA	Dog	Women prisoners and serious mental illness	Single-case design	19	11	8	GAD-7, PCL y PHQ-9	30	8	8
14 Holman, Wilkerson, Ellmo & Skirius (2020b) [30]	USA	Dog	Women prisoners and serious mental illness	Single-case design	6	-	-	GAD-7	30	8	8
15 Hunt et al. (2014) [31]	USA	Dog	Without diagnosis	RCT	107	-	-	STAI-S	20	-	-
16 Johnson et al. (2018) [32]	USA	Horse	PTSD/Brain injury	RCT (waiting-list control)	29	14	15	PCL-M	40	6	6
17 Johnson et al. (2021) [33]	USA	Horse	PTSD/Brain Injury	RCT	20	-	-	THR (Ad hoc questionnaire)	60	6	6
18 Kang et al. (2018) [34]	South Korea	Horse	Internet gaming disorder	Experimental	30	15	15	K-ECRS	60	14	1
19 Kiesewetter, J., et al. (2023) [35]	Germany	Dog	Pain	Quasi-experimental (controlled)	56	29	26	STAIC	180	12	12
20 López-Fernández, E et al. (2023) [36]	Spain	Dog	Paediatrics	Quasi-experimental (pre-experimental)	61	-	-	m-YPAS	38	74	-
21 Marchand, WR., et al. (2023) [37]	USA	Horse	Substance abuse, PTSD	Pilot study (within-subject)	94	-	-	STAI	-	1–6	-
22 Mattila-Rautiainen, S., et al. (2023) [38]	Finland	Horse	Chronic low back pain	Pilot study (within-subject)	22	-	-	R-BDI	10–30	12	12
23 McCullough et al. (2018) [39]	USA	Dog	Paediatric oncology	RCT (multicentre)	106	46	60	STAI, STAI-CH, heart rate and respiratory rate metre.	10–12	16	16
24 Monroe et al. (2019) [40]	USA	Horse	PTSD	Quasi-experimental	38	-	-	Burns Anxiety Inventory (BAI)	90	8	1
25 Mulvaney-Roth et al. (2023) [41]	USA	Dog	Behaviour and paediatrics	Experimental	42 adults/12 children	14/6	28/6	SAS	5–15/5–30	-	-
26 Nepps et al. (2014) [42]	USA	Dog	PTSD/Brain injury	Experimental	218	84	134	BAI, spit collection, heart rate and respiration rate metre	60	1	-
27 Perez et al. (2019) [43]	Canada	Dog	Paediatrics	Pilot Study	21	-	-	Ad hoc questionnaire	20–60	1	1
28 Romaniuk et al. (2018) [44]	USA	Horse	PTSD	Longitudinal observational	47	-	-	PCL-5, DASS-21	-	5	-
29 Shelton, A. M. (2022) [45]	USA	Horse	Adult children of divorce	Qualitative (IPA)	3	-	-	Semi-structured interview via Zoom	Variable	Variable	4
30 Silva & Osório (2018) [46]	Brazil	Dog	Paediatric oncology	Quasi-experimental	24	-	-	Children: Child Stress Symptom Inventory, BRUMS, heart rate. Carers: STAI	30	3	4
31 Vegue et al. (2021) [47]	Spain	Dog	Dementia	RCT (multicentre)	334	-	-	CSDD-7 (anxiety subtest)	45	32	32

Abbreviations: PTSD, Post-Traumatic Stress Disorder; RCT, Randomised Controlled Trial; STAI, State–Trait Anxiety Inventory; PCL, PTSD Checklist; GAD-7, Generalized Anxiety Disorder scale; BAI, Burns Anxiety Inventory; PSS, Perceived Stress Scale; DASS-21, Depression Anxiety Stress Scale–21; PHQ-9, Patient Health Questionnaire.

## Data Availability

No new data were created or analysed in this study.
